# Self-Perceived Fitness in Young Athletes: Associations with Anthropometric Markers and Lipid Profile as Cardiometabolic Risk Factors—COR-SCHOOL Study

**DOI:** 10.3390/jfmk10020175

**Published:** 2025-05-14

**Authors:** Alvaro Pano-Rodriguez, Saül Aixa-Requena, Abraham Batalla-Gavaldà, Jose Vicente Beltran-Garrido, Isaac López-Laval, Vicenç Hernández-González, Carme Jové-Deltell, Enric Conesa-Milian, Joaquin Reverter-Masia

**Affiliations:** 1 Faculty of Education, Psychology and Social Work, Department of Specific Didactics, University of Lleida, 25003 Lleida, Spain; saul.aixa@udl.cat (S.A.-R.); vicenc.hernandez@udl.cat (V.H.-G.); carme.jove@udl.cat (C.J.-D.); enric.conesa@udl.cat (E.C.-M.); joaquim.reverter@udl.cat (J.R.-M.); 2 Human Movement Research Group (RGHM), University of Lleida, 25003 Lleida, Spain; 3 University School of Health and Sport (EUSES), Universitat Rovira i Virgili, 43870 Amposta, Spain; a.batalla@euseste.es; 4 Department of Education and Specific Didactics, Faculty of Humanities and Social Sciences, Universitat Jaume I, 12071 Castellón de la Plana, Spain; 5 Physical Exercise and Performance Research Group, Department of Education Sciences, School of Humanities and Communication Sciences, Universidad Cardenal Herrera—CEU, CEU Universities, 12006 Castellón de la Plana, Spain; jose.beltrangarrido@uchceu.es; 6 Faculty of Health and Sport Science, Department of Physiatry and Nursing, University of Zaragoza, 22001 Huesca, Spain; isaac@unizar.es

**Keywords:** physical fitness perception, anthropometric variables, biochemical parameters, sex differences, youth athletes

## Abstract

**Objective**: This study analyzed the relationship between self-perceived physical fitness and anthropometric and biochemical variables in young athletes from extracurricular sports programs in northeastern Spain. **Methods**: A cross-sectional design was used with a sample of 673 young athletes. Data collection included self-reported physical fitness and objective anthropometric and biochemical measurements. The analysis explored associations between perceived fitness dimensions and physical/biochemical variables, with attention to sex differences. **Results**: Fat mass showed significant inverse associations with all perceived fitness dimensions: general fitness (OR = 0.62, 95% CI [0.41, 0.94]), cardiorespiratory fitness (OR = 0.56, 95% CI [0.37, 0.83]), muscular strength (OR = 0.61, 95% CI [0.41, 0.91]), speed/agility (OR = 0.59, 95% CI [0.39, 0.88]), and flexibility (OR = 0.57, 95% CI [0.39, 0.84]). Higher fat mass was consistently linked to lower perceived fitness. HDL levels were positively associated with general (OR = 1.40, 95% CI [1.13, 1.74]) and cardiorespiratory fitness (OR = 1.32, 95% CI [1.07, 1.62]), while LDL levels showed no significant effect (*p* > 0.05). Sex differences emerged for general fitness (OR = 0.52, 95% CI [0.33, 0.82]) and flexibility (OR = 0.51, 95% CI [0.33, 0.78]), favoring boys, but no differences were found for cardiorespiratory fitness, muscular strength, or speed/agility (*p* > 0.05). This suggests that shared athletic environments may reduce typical sex-based disparities. **Conclusions**: Our findings emphasize the importance of considering both anthropometric and biochemical variables when evaluating perceived fitness in youth athletes. Regular athletic engagement may buffer sex-based differences in fitness perception.

## 1. Introduction

Physical fitness during childhood and adolescence is a key factor for lifelong health, as it is closely linked to physical, psychological, and social well-being [1,2]. Numerous studies have demonstrated that perceived physical fitness at an early age is associated with various fitness and health outcomes [3], emphasizing the importance of continuous evaluation and monitoring. For instance, higher levels of self-perceived fitness during childhood have been linked to better physical literacy, which in turn correlates with improved health behaviors and physical activity levels [4]. Furthermore, adolescents who report positive perceptions of their physical fitness tend to experience fewer psychosomatic health complaints, highlighting the relevance of fostering positive fitness perceptions early in life [5].

Self-reporting methodologies have been widely used as valid and reliable tools to assess multiple dimensions of physical fitness, including general fitness, cardiorespiratory fitness, muscular strength, speed/agility, and flexibility [3]. This procedure not only allows for a comprehensive assessment of fitness levels but also facilitates the comparison of self-perceived fitness across diverse populations and settings. However, the primary value of self-reported fitness scales also lies in their ability to capture subjective experiences and perceptions, which may not always align with objectively measured fitness and health parameters. This discrepancy between perceived and actual fitness is particularly relevant in adolescent populations, where social and psychological factors may influence self-assessment [6].

While many studies have focused on objective determinants of physical fitness, less attention has been given to subjective factors influencing self-perception, particularly among pediatric and adolescent populations. This gap is critical because understanding the factors that shape self-perception can inform interventions aimed at promoting realistic and positive self-assessments, which, in turn, may enhance physical activity engagement and overall health outcomes. Additionally, self-perceived fitness has been associated with psychological constructs such as self-esteem and body image [7], which further underscores the need for integrative approaches when evaluating fitness in young populations.

Sex differences in perceived physical fitness also constitute a relevant topic in current research. Previous studies have suggested that girls tend to perceive higher levels of flexibility compared to boys, while boys often report greater levels of muscular strength and agility [8]. Furthermore, boys consistently demonstrate higher self-confidence in their physical abilities, including strength and sport competence, compared to girls [9]. This difference is partly attributed to social and psychological factors that influence how adolescents evaluate their physical capabilities, with girls showing lower self-confidence and greater insecurity related to physical self-perception [9]. Additionally, anthropometric factors such as body mass index (BMI), fat mass, and lean body mass may significantly influence self-perceived fitness. In fact, self-perceived body image has been shown to correlate positively with fat mass and BMI, especially among females, highlighting the influence of adiposity on physical self-worth [10]. Moreover, muscular fitness has been found to play a more significant role in boys’ physical self-perception, where absolute muscular strength positively correlates with perceived strength and overall physical self-worth [11].

Likewise, biochemical parameters such as high-density lipoprotein (HDL) and low-density lipoprotein (LDL) levels may also play an important role, especially in the context of cardiovascular health. Elevated LDL levels have been consistently linked to increased cardiovascular risk, as oxidized LDL contributes to the formation of atherosclerotic plaques and promotes endothelial inflammation. In contrast, HDL exerts cardioprotective effects through its anti-inflammatory, antioxidative, and antithrombotic properties, helping maintain endothelial integrity and cholesterol efflux from artery walls [12,13]. Thus, given the well-established role of HDL and LDL levels in cardiovascular risk assessment, these parameters may serve as valuable variables to compare self-perceived cardiovascular health with objectively measured health status [14,15].

Despite these findings, the relationship between anthropometric and biochemical variables with subjective fitness perception remains unclear, as previous studies have produced inconsistent results. Therefore, the present study aims to fill this gap by analyzing self-reported physical fitness scores according to sex, age, BMI, fat mass percentage, lean body mass, and HDL and LDL levels in primary school students. The findings are expected to provide a better understanding of the determinants of self-perceived physical fitness during childhood and adolescence, which may help design effective intervention strategies to improve health and well-being in this population.

In this context, the current study seeks to clarify how various anthropometric and biochemical indicators are associated with self-perceived physical fitness among primary school children. Specifically, the research question guiding this inquiry is as follows: To what extent do sex, age, body composition parameters (BMI, fat mass, and lean body mass), and lipid profile (HDL-C and LDL-C levels) predict self-perceived physical fitness in a population of young athletes? By addressing this question, this study aims to contribute to the understanding of how subjective fitness perceptions relate to objective health markers, providing valuable insights for health promotion during childhood and adolescence.

## 2. Materials and Methods

### 2.1. Study Design

This study followed a cross-sectional design aimed at analyzing the relationship between self-perceived physical fitness and anthropometric and biochemical variables in primary school students. A cross-sectional study involves the observation of a population at a single point in time, allowing for the identification of patterns and relationships among variables without inferring causality. This design was selected because it is particularly well suited for estimating prevalence and exploring potential associations in large, naturally occurring populations such as school settings [16]. The study was conducted within the framework of the COR-SCHOOL Project, an initiative focused on assessing cardiometabolic health indicators among school-aged children and adolescents.

### 2.2. Participants

This study included 673 young athletes (56.2% boys) aged 6 to 12 years recruited from extracurricular sports programs located in the provinces in northeastern Spain. The study was conducted in this region based on the research team’s institutional affiliation and established collaborations with local educational and sports organizations. This location was also chosen for its practical accessibility and sociodemographic diversity, offering both urban and rural settings aligned with the objectives of the COR-SCHOOL Project. Participants were selected through a stratified random sampling approach to ensure representativeness across different educational institutions. Inclusion criteria comprised enrollment in primary education, informed consent from parents or guardians, and the ability to complete the self-reported fitness questionnaire. Exclusion criteria included any medical condition that could significantly influence physical activity, as reported by the participants themselves. The study was conducted in accordance with the ethical guidelines of the Declaration of Helsinki [17] and was approved by the Ethics Committee for Clinical Research of the Catalan Council (30/CEICGC/2020).

### 2.3. Outcomes

#### 2.3.1. Self-Perceived Physical Fitness

The self-perceived physical fitness was assessed using the International Fitness Information System (IFIS), a validated self-report tool that evaluates general fitness, cardiorespiratory fitness, muscular strength, speed/agility, and flexibility [18,19]. The IFIS has demonstrated adequate reliability and validity for assessing self-perceived health-related fitness in Spanish children, supporting its use in epidemiological studies involving young populations [20].

Data collection was carried out electronically using electronic tablets, ensuring that responses were directly recorded and securely stored in a dedicated database for later processing and analysis. Following the data analysis, each participant received a personalized summary report with their individual results.

#### 2.3.2. Anthropometric Variables

Anthropometric measurements were conducted following the standards of the International Society for the Advancement of Kinanthropometry (ISAK) [21]. Body mass was measured using a digital scale (Seca 877, Hamburg, Germany; accuracy ± 0.1 kg), and height was assessed with a portable stadiometer (Seca 213, Hamburg, Germany; accuracy ± 0.1 cm) to calculate the BMI by dividing body mass in kilograms by the square of height in meters (kg/m^2^). Skinfold thickness was measured using a calibrated skinfold caliper (Harpenden, Baty International, Burgess, UK; accuracy ± 0.2 mm) at three standard anatomical sites: triceps, subscapular, and supraspinale. All measurements were performed on the right side of the body, in accordance with the standardized procedures of the International Society for the Advancement of Kinanthropometry (ISAK). Each measurement was taken twice, and a third measurement was taken if the first two differed by more than 5%. The final value used was the average of the closest two readings. Assessments were conducted by trained personnel certified at a minimum of ISAK Level 1. A non-elastic anthropometric tape (Lufkin W606PM, Apex Tool Group, Sparks, MD, USA) was used to measure girths when required. Estimates of fat and lean body mass percentages were derived using ISAK-approved population-specific equations appropriate for the age and characteristics of the participant’s lean body mass [21].

#### 2.3.3. Biochemical Variables

Biochemical parameters, including HDL and LDL levels, were analyzed from fasting blood samples using enzymatic colorimetric methods [22]. Blood samples were collected by qualified healthcare personnel and processed under standardized laboratory conditions to ensure accuracy and reproducibility. LDL-C was estimated using the Friedewald formula (LDL-C = total cholesterol − HDL-C − [triglycerides/5]), which is commonly used for clinical and research purposes and has been validated in pediatric populations under fasting conditions [23]. Total cholesterol and serum triglycerides were not included in the present analysis, as the study focused specifically on the roles of HDL-C and LDL-C in relation to self-perceived fitness.

To minimize the potential effects of recent strenuous physical activity on serum lipid levels [24], parents or legal guardians, as well as the coordinators of the extracurricular sports programs, were instructed to ensure that the participants avoided intense physical exercise during the 24 h prior to blood sampling. This recommendation was provided in writing as part of the study instructions distributed before the health assessment.

### 2.4. Statistical Analysis

To assess data normality, the Kolmogorov–Smirnov test, Q-Q plot, and histogram plot of residuals were used, as they are particularly suitable for detecting deviations from normality in large samples. The combination of visual (Q-Q plot and histograms) and statistical methods ensured a robust assessment of the distributional assumptions required for subsequent analyses. Given the sample size (N = 673), the Kolmogorov–Smirnov test was deemed appropriate. Group comparisons were performed using Student’s *t*-test for normally distributed variables and the Mann–Whitney U test for non-normally distributed variables, providing appropriate sensitivity depending on data characteristics. Categorical variables were analyzed using the chi-square test. Post hoc analyses using adjusted residuals were conducted to identify specific differences in the distribution of self-reported IFIS dimensions between sexes, allowing for a more detailed understanding of where there were group differences.

Generalized linear models (GLMs) were used to estimate odds ratios (ORs) and 95% confidence intervals (CIs) for self-reported IFIS dimension scores, stratified by sex and adjusted for age, BMI, fat mass percentage, lean body mass, HDL levels, and LDL levels. GLMs were selected due to their flexibility in modeling non-normally distributed outcome variables and their capacity to include covariates, making them suitable for the exploratory nature of this study [25]. The different dimensions of the IFIS scale (i.e., general fitness, cardiorespiratory fitness, muscular strength, speed/agility, and flexibility) were included as dependent variables in separate GLMs. Since only one categorical variable (sex) with two levels was included, no multiple-comparison adjustments were applied.

Model fit was evaluated using log likelihood, Akaike Information Criterion (AIC), and Bayesian Information Criterion (BIC), which are standard indices to assess and compare the relative quality of statistical models [25]. Statistical significance was set at α < 0.05. Data are reported as mean (SD), median (Mdn) with interquartile range (IQR: Q1–Q3), or frequency, depending on the variable type.

All analyses were performed using JAMOVI for Mac [26] (2025) (version 2.6.44) and the GAMLj module for generalized linear modeling [27] (Table 1).

## 3. Results

### 3.1. Sex Differences

Statistically significant differences between sexes were reported for fat mass percentage (*p* < 0.001), lean body mass (*p* < 0.001), HDL (*p* < 0.001), LDL (*p* < 0.001), CRF (*p* = 0.026), and flexibility (*p* < 0.001) scores.

The post hoc test using adjusted residuals revealed that the proportion of women reporting “very good” flexibility was significantly higher than expected (z = 2.83, *p* < 0.01), whereas the proportion of men in the same category was significantly lower than expected (z = −2.73, *p* < 0.01). No significant differences were observed in the remaining flexibility categories (very poor, poor, average, and good), as the adjusted residuals did not exceed the significance threshold (|z| < 1.96). These findings suggest that women tend to perceive themselves as having higher flexibility levels compared to men.

However, for the self-reported cardiorespiratory fitness, the post hoc results revealed no significant differences between sexes, as the adjusted residuals for all categories (very poor, poor, average, good, and very good) remained below the significance threshold (|z| < 1.96). These findings suggest that men and women perceive their cardiorespiratory fitness levels similarly, with no clear sex-based trends in self-reported IFIS-CRF scores.

### 3.2. Fitness Perception

**Figure 1:**
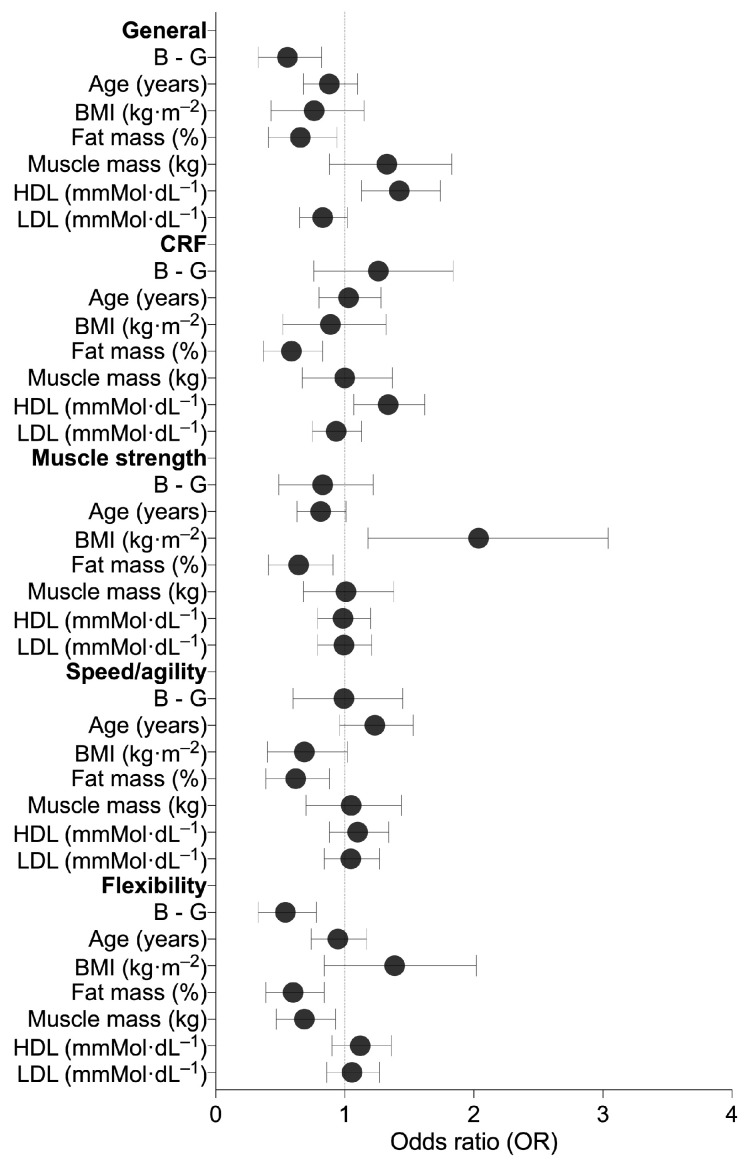
Forest plot showing the odds ratios (OR) for each dimension of the International Fitness Scale (IFIS), stratified by sex. Black dots represent the estimated ORs, and horizontal lines indicate the 95% confidence intervals. B: boys; G: girls; CRF: cardiorespiratory fitness; BMI: body mass index; HDL: high-density lipoprotein cholesterol; LDL: low-density lipoprotein cholesterol.

The parameter estimates (coefficients) of the generalized linear models for the different dimensions of the IFIS scale are shown in the Supplementary Materials File S1. The forest plot of the odds ratios of the different dimensions of the IFIS scale is shown in Figure 1.

#### 3.2.1. General Fitness

A statistically significant effect of the sex variable (b = −0.66, z = −2.78, *p* = 0.005) was observed. Boys had a lower probability of reporting higher self-perceived general fitness scores compared to girls (OR = 0.52; 95% CI [0.33, 0.82]); see Figure 1.

A statistically significant effect of the fat mass variable (b = −0.48, z = −2.25, *p* = 0.024) was observed: the higher the fat mass levels, the lower the probability of having higher self-reported general fitness scores (OR = 0.62; 95% IC [0.41, 0.94]); see Figure 1. No significant interaction was observed with the sex variable.

A statistically significant effect of the HDL variable (b = 0.34, z = 3.05, *p* = 0.002) was observed: the higher the levels of HDL, the higher the probability of having higher self-reported general fitness scores (OR = 1.40; 95% IC [1.13, 1.74]); see Figure 1. No significant interaction was observed with the sex variable.

#### 3.2.2. Cardiorespiratory Fitness

A statistically significant effect of the fat mass variable (b = −0.58, z = −2.85, *p* = 0.004) was observed: the higher the fat mass levels, the lower the probability of having higher self-reported cardiorespiratory fitness scores (OR = 0.56; 95% IC [0.37, 0.83], Figure 1). No significant interaction was observed with the sex variable.

A statistically significant effect of the HDL variable (b = 0.27, z = 2.56, *p* = 0.010) was identified: the higher the levels of HDL, the higher the probability of having higher self-reported cardiorespiratory fitness scores (OR = 1.32 95%; IC [1.07, 1.62], Figure 1). No significant interaction was observed with the sex variable.

#### 3.2.3. Muscular Strength

A statistically significant effect of the BMI variable (b = 0.64, z = 2.65, *p* = 0.008) was observed: the higher the levels of BMI, the higher the probability of having higher self-reported muscular strength scores (OR = 1.89; 95% IC [1.18, 3,04], Figure 1). No significant interaction was observed with the sex variable.

A statistically significant effect of the fat mass variable (b = −0.49, z = −2.43, *p* = 0.015) was found: the higher the fat mass levels, the lower the probability of having higher self-reported muscular strength scores (OR = 0.61; 95% IC [0.41, 0.91], Figure 1). No significant interaction was observed with the sex variable.

#### 3.2.4. Speed/Agility

A statistically significant effect of the fat mass variable (b = −0.54, z = −2.60, *p* = 0.009) was observed: the higher the fat mass levels, the lower the probability of having higher self-reported speed/agility scores (OR = 0.59; 95% IC [0.39, 0.88], Figure 1). No significant interaction was observed with the sex variable.

#### 3.2.5. Flexibility

A statistically significant effect of the sex variable (b = −0.68, z = −3.08, *p* = 0.002) was found. Boys had a lower probability of reporting higher self-perceived flexibility scores compared to girls (OR = 0.51; 95% CI [0.33, 0.78], Figure 1).

A statistically significant effect of the fat mass variable (b = −0.56, z = −2.87, *p* = 0.004) was observed: the higher the fat mass levels, the lower the probability of having higher self-reported flexibility scores (OR = 0.57; 95% CI [0.39, 0.84], Figure 1). No significant interaction was observed with the sex variable.

A statistically significant effect of the lean body mass variable (b = −0.41, z = −2.36, *p* = 0.018) was observed: the higher the levels of lean body mass, the lower the probability of having higher self-reported flexibility scores (OR = 0.66; 95% CI [0.47, 0.93], Figure 1). No significant interaction was observed with the sex variable.

## 4. Discussion

The aim of the present study was to analyze the relationship between self-perceived physical fitness and anthropometric and biochemical variables in young athletes participating in extracurricular sports activities in northeastern Spain. The primary findings highlight significant associations between several dimensions of self-perceived fitness and specific anthropometric and biochemical variables, with some notable differences between sexes. These findings provide valuable insights into how physical self-perception is shaped by both physical and biological factors during childhood and adolescence.

### 4.1. Association Between Body Composition and Self-Perceived Physical Fitness

One of the most relevant findings of this study was the significant association between fat mass and self-perceived physical fitness across all fitness dimensions (i.e., general fitness, cardiorespiratory fitness, muscular strength, speed/agility, and flexibility). Specifically, higher fat mass levels were consistently associated with lower self-perceived fitness scores, suggesting that adiposity negatively influences how young athletes perceive their physical abilities. These results are consistent with previous studies that have reported similar associations between body composition and self-perceived fitness, emphasizing the impact of excess body fat on physical self-concept [10,28].

Interestingly, the inverse association between fat mass and perceived fitness was strongest for cardiorespiratory fitness, with decreasing magnitudes also observed for flexibility, speed/agility, muscular strength, and general fitness. This pattern could be explained by the biomechanical limitations imposed by higher fat mass [29], which may reduce performance in activities requiring rapid and forceful movements [11]. Moreover, it is noteworthy that the relationship between adiposity and perceived flexibility was less consistent, suggesting that other factors, such as joint mobility and muscle elasticity, may play a more decisive role in flexibility perception [30].

A relevant aspect of our findings is the significant positive relationship between BMI and self-perceived muscular strength, which may appear contradictory given the negative effect of fat mass on muscular strength perception. This apparent paradox can be explained by considering that BMI does not distinguish between fat mass and lean mass [31], implying that muscle development in adolescents may contribute to higher BMI without necessarily indicating increased adiposity.

In this context, it is plausible that young athletes with greater muscle development perceive themselves as physically stronger, leading to higher scores in self-reported muscular strength. This underscores the importance of carefully interpreting BMI as an indirect indicator of body composition, particularly in physically active populations where lean mass may represent a significant proportion of total body weight [32].

### 4.2. Biochemical Parameters and Self-Perceived Fitness

Regarding the association between biochemical parameters and self-perceived fitness, the results indicated a positive relationship between HDL levels and self-reported general and cardiorespiratory fitness. This finding suggests that favorable lipid profiles may enhance perceptions of cardiovascular health, as HDL has been linked to improved endothelial function and reduced cardiovascular risk through its anti-inflammatory, antioxidative, and vasodilatory effects [33]. In contrast, elevated LDL levels—typically linked to increased atherogenic risk—did not significantly influence self-perceived fitness scores. This finding reinforces the idea that subjective perceptions of fitness may not fully capture underlying cardiovascular risk factors. In this regard, emerging evidence indicates that the association between lipid profiles and perceived health is likely shaped more by lifestyle habits, physical activity levels, and overall cardiovascular fitness than by isolated biochemical markers [34].

### 4.3. General Fitness: Effect of Sex and Fat Mass

The results of our study show a significant association between sex and self-perceived general fitness, with boys having a lower probability of reporting high scores compared to girls. The fact that both sexes of our sample participate in extracurricular sports activities likely fosters similar physical competencies and self-perceptions [35,36], as the active lifestyle shared by all participants seems to contribute to minimizing the typical sex differences commonly observed in non-athletic populations [37]. Additionally, the positive self-perception observed among girls in this study, particularly regarding general physical fitness, might reflect a growing emphasis on inclusive and empowering sports environments that encourage girls to develop self-esteem in their physical abilities [38,39]. This context may partly explain the divergence from previous studies that reported lower self-perception of fitness among girls [37,40], underscoring the importance of considering sample characteristics when interpreting self-perception outcomes.

Notably, no significant interaction was observed between sex and fat mass, suggesting that the negative impact of adiposity is consistent across sexes.

### 4.4. Sex Differences in Flexibility Perception

The observed differences between sexes in flexibility align with previous research indicating that girls consistently demonstrate higher levels of flexibility compared to boys. This difference is supported by studies that highlight sex-based anatomical and physiological factors, such as greater joint mobility and increased tissue elasticity among females, which may be influenced by higher production of relaxin [41]. Additionally, social and cultural norms that promote flexibility-related activities in girls from a young age may further reinforce these differences [9]. Moreover, research has shown that girls tend to perceive themselves as more flexible compared to boys, further emphasizing the role of sex differences in flexibility perception [9].

These sex disparities in flexibility have practical implications for physical education and training programs. Recognizing the inherent variability in flexibility between boys and girls is essential for designing tailored assessments and interventions that accurately reflect their physical capabilities. A more individualized approach could help foster a positive and realistic perception of physical fitness among both sexes.

### 4.5. Sex Differences in Cardiorespiratory Fitness Perception

An intriguing observation in this study was the lack of significant differences in self-perceived cardiorespiratory fitness between boys and girls, despite evidence suggesting that boys typically exhibit greater objective cardiorespiratory capacity [37]. This finding diverges from previous research that often reports a higher self-perceived cardiorespiratory fitness among boys, likely due to social and psychological factors that influence self-assessment, including societal expectations and sex norms [42,43].

Nevertheless, our study’s unique context may explain this divergence. Unlike general adolescent populations, our sample consists exclusively of young athletes who regularly participate in structured sports activities within sports clubs. This shared training environment likely contributes to a leveling of perceived cardiorespiratory fitness between boys and girls [44], as both groups develop similar physical competencies and self-perceptions through consistent athletic practice. Such a context may mitigate the typical sex differences observed in non-athletic populations [37], underscoring the importance of considering sample characteristics when interpreting self-perception outcomes.

### 4.6. Strengths and Limitations

This study presents several strengths, including a substantial sample size and the combination of objective anthropometric and biochemical data with subjective assessments of physical fitness. Furthermore, employing rigorous statistical methods, such as generalized linear models, guarantees a precise interpretation of the findings. However, its cross-sectional design limits causal inferences, and the reliance on self-reported data may introduce biases related to social desirability or subjective interpretation. Moreover, since the sample was drawn from a single geographic region in Spain, the external validity of the findings may be limited when extrapolating results to other populations or cultural contexts. Additionally, although this study provides relevant insights based on self-perceived physical fitness, the absence of objective fitness assessments (e.g., cardiorespiratory endurance, muscular strength, or agility tests) represents a limitation. Future studies should incorporate such measures to validate and complement subjective reports and to better capture the multidimensional nature of physical fitness in youth populations.

### 4.7. Practical Implications and Future Research

The results of this study have practical implications for designing interventions aimed at enhancing self-perception of physical fitness in young athletes. An integrated approach that addresses both physical and psychological aspects is essential for fostering realistic and positive self-assessments, which can ultimately promote healthier lifestyle choices. Recent research emphasizes the importance of combined interventions that consider both fitness levels and self-perception as critical determinants of long-term health outcomes [45]. Future research should focus on longitudinal studies to explore causal relationships and interventions that target the interplay between physical attributes and self-perception. Additionally, examining how these relationships evolve across different developmental stages could provide valuable insights for tailored intervention strategies.

## 5. Conclusions

This study demonstrated significant associations between self-perceived physical fitness and anthropometric and biochemical variables in young athletes, with notable differences between boys and girls. The results showed that higher fat mass was consistently linked to lower self-perceived fitness dimensions. Additionally, HDL levels were positively associated with self-reported general and cardiorespiratory fitness, while LDL levels did not significantly impact perception.

Sex differences emerged in perceptions of general fitness and flexibility, with girls reporting higher self-perceived fitness despite comparable athletic involvement. This could reflect the growing emphasis on inclusive sports environments that foster confidence and self-esteem among female athletes. The lack of significant sex differences in perceived cardiorespiratory fitness, despite boys generally having higher objective capacity, suggests that regular athletic participation may help equalize self-perception between sexes.

## Figures and Tables

**Table 1:** Descriptive characteristics of the study population.

Variable	All (n = 673)	Boys (n = 402)	Girls (n = 271)	*p* Values
**Age (years) ^a^**	11.63 ± 1.65	11.65 ± 1.63	11.61 ± 1.68	0.571
**BMI (kg·m^−2^) ^a^**	19.74 ± 3.20	19.59 ± 3.26	19.95 ± 3.09	0.052
**Fat mass (%) ^a^**	23.22 ± 6.19	21.38 ± 6.47	25.60 ± 4.90	**<0.001**
**Lean body mass (kg) ^b^**	33.94 ± 8.08	35.01 ± 8.68	32.56 ± 7.02	**<0.001**
**HDL (mmMol·dL^−1^) ^b^**	47.24 ± 15.36	44.20 ± 15.32	51.80 ± 14.29	**<0.001**
**LDL (mmMol·dL^−1^) ^b^**	71.00 ± 27.04	66.58 ± 26.40	77.62 ± 26.68	**<0.001**
**General ^c^**	1/10/107/375/180	1/6/42/146/79	0/4/42/146/79	0.740
**CRF ^c^**	7/31/210/306/119	3/14/112/199/77	4/17/98/107/45	**0.026**
**Muscular strength ^c^**	3/20/186/334/130	2/8/114/198/80	1/12/72/136/50	0.450
**Speed/agility ^c^**	3/26/166/279/199	1/17/95/157/132	2/9/71/122/67	0.156
**Flexibility ^c^**	36/143/266/159	23/95/173/86/25	13/48/93/73/44	**<0.001**

Values in bold indicate statistically significant results (*p* < 0.05). Abbreviations: BMI: body mass index, HDL: high-density lipoprotein, LDL: low-density lipoprotein, CRF: cardiorespiratory fitness. ^a^ Data are presented as median (IQR), and differences between boys and girls were examined using an independent-sample Mann–Whitney U test. ^b^ Data are presented as mean (SD), and differences between boys and girls were examined using analysis of variance. ^c^ Data are presented as frequency (%), and differences between boys and girls were examined using an independent-sample chi-square test.

## Data Availability

The data presented in this study are available upon request from the corresponding author due to ethical restrictions related to the protection of sensitive information and privacy of underage participants, in accordance with institutional and data protection regulations.

## Supplementary Materials

The following supporting information can be downloaded at: https://www.mdpi.com/article/10.3390/jfmk10020175/s1, File S1: Generalized linear models for the different dimensions of the IFIS scale.
