# Supplementary material for: Self-Perceived Fitness in Young Athletes: Associations with Anthropometric Markers and Lipid Profile as Cardiometabolic Risk Factors—COR-SCHOOL Study
Source: J Funct Morphol Kinesiol. 2025 May 14;10(2):175. doi: 10.3390/jfmk10020175 (PMC12101379; doi:10.3390/jfmk10020175)
Supplement: Supplementary file 1 [file jfmk-10-00175-s001.zip › jfmk-3613466-supplementary.pdf]

File S1. Generalized linear models for the different dimensions of the IFIS scale are shown in the supplementary materials.

Generalized linear model for the Overall dimension of the IFIS scale.

Effect	Estimate	SE	OR	Lower 95% CI	Upper 95% CI	z	p
Male - Female	-0.66	0.24	0.52	0.33	0.82	-2.78	0.005
Age	-0.15	0.12	0.86	0.68	1.10	-1.20	0.231
BMI	-0.35	0.25	0.71	0.43	1.15	-1.41	0.158
Fat mass (%)	-0.48	0.21	0.62	0.41	0.94	-2.25	0.024
Muscle Mass (kg)	0.24	0.19	1.27	0.88	1.83	1.26	0.206
HDL pre	0.34	0.11	1.40	1.13	1.74	3.05	0.002
LDL pre	-0.20	0.11	0.82	0.65	1.02	-1.78	0.076

Generalized linear model for the Cardiorespiratory dimension of the IFIS scale.

Effect	Estimate	SE	OR	Lower 95% CI	Upper 95% CI	z	p
Male - Female	0.17	0.23	1.18	0.76	1.84	0.75	0.453
Age	0.01	0.12	1.01	0.80	1.28	0.12	0.903
BMI	-0.19	0.24	0.83	0.52	1.32	-0.79	0.427
Fat mass (%)	-0.58	0.20	0.56	0.37	0.83	-2.85	0.004
Muscle Mass (kg)	-0.04	0.18	0.96	0.67	1.37	-0.23	0.822
HDL pre	0.27	0.11	1.32	1.07	1.62	2.56	0.010
LDL pre	-0.09	0.11	0.92	0.75	1.13	-0.83	0.407

Generalized linear model for the Muscular strength dimension of the IFIS scale.

Effect	Estimate	SE	OR	Lower 95% CI	Upper 95% CI	z	p
Male - Female	-0.25	0.23	0.78	0.49	1.22	-1.10	0.273
Age	-0.23	0.12	0.80	0.63	1.01	-1.90	0.057
BMI	0.64	0.24	1.89	1.18	3.04	2.65	0.008
Fat mass (%)	-0.49	0.20	0.61	0.41	0.91	-2.43	0.015
Muscle Mass (kg)	-0.04	0.18	0.97	0.68	1.38	-0.19	0.847
HDL pre	-0.03	0.11	0.97	0.79	1.20	-0.25	0.799
LDL pre	-0.02	0.11	0.98	0.79	1.21	-0.19	0.846

Generalized linear model for the Speed/agility dimension of the IFIS scale.

Effect	Estimate	SE	OR	Lower 95% CI	Upper 95% CI	z	p
Male - Female	-0.07	0.23	0.93	0.60	1.45	-0.32	0.750
Age	0.19	0.12	1.21	0.96	1.53	1.61	0.106
BMI	-0.45	0.24	0.64	0.40	1.02	-1.87	0.061
Fat mass (%)	-0.54	0.21	0.59	0.39	0.88	-2.60	0.009
Muscle Mass (kg)	0.01	0.18	1.01	0.70	1.44	0.04	0.972
HDL pre	0.08	0.11	1.08	0.88	1.34	0.74	0.461
LDL pre	0.03	0.11	1.03	0.84	1.27	0.30	0.763

Generalized linear model for the Flexibility dimension of the IFIS scale.

Effect	Estimate	SE	OR	Lower 95% CI	Upper 95% CI	z	p
Male - Female	-0.68	0.22	0.51	0.33	0.78	-3.08	0.002
Age	-0.07	0.11	0.93	0.74	1.17	-0.63	0.530
BMI	0.26	0.22	1.30	0.84	2.02	1.19	0.236
Fat mass (%)	-0.56	0.19	0.57	0.39	0.84	-2.87	0.004
Muscle Mass (kg)	-0.41	0.17	0.66	0.47	0.93	-2.36	0.018
HDL pre	0.10	0.10	1.10	0.90	1.36	0.95	0.343
LDL pre	0.04	0.10	1.04	0.86	1.27	0.41	0.681
